# Supplementary material for: Remote Adipose Tissue-Derived Stromal Cells of Patients with Lung Adenocarcinoma Generate a Similar Malignant Microenvironment of the Lung Stromal Counterpart
Source: J Oncol. 2023 Jan 24;2023:1011063. doi: 10.1155/2023/1011063 (PMC9889152; doi:10.1155/2023/1011063)
Supplement: Supplementary Materials — Clinical characteristics of adipose tissue-derived MSC samples and the apoptotic rate of the cell cultures treated with MSC-derived supernatants are reported in the supplementary table 1a-b and Figure 1a-b, respectively. [file 1011063.f1.zip › Table MSC patients.docx]

**Supplementary Table 1a-b.**

| **Code** | **Gender** | **Age (years)** |
| --- | --- | --- |
| 28M | F | 53 |
| 42M | M | 78 |
| 44M | M | 54 |
| 36M | M | 41 |

1. Autologous mediastinal Adipose tissue derived MSC isolated from patients with hamartocondroma
2. Autologous mediastinal Adipose tissue derived MSC isolated from patients with lung adenocarcinoma

| **Code** | **Gender** | **Age (years)** | **Lung Adenocarcinoma Histology (grade)** |
| --- | --- | --- | --- |
| 32M | M | 72 | G2pT1a |
| 35M | M | 68 | G2 pT1b pN0MxIa |
| Adeno 1 | M | 68 | pT1b pN0Ia |
| 55M | F | 68 | G2pT1bpN0Ia |
