# Supplementary figures and images for: Remote Adipose Tissue-Derived Stromal Cells of Patients with Lung Adenocarcinoma Generate a Similar Malignant Microenvironment of the Lung Stromal Counterpart
Source: J Oncol. 2023 Jan 24;2023:1011063. doi: 10.1155/2023/1011063 (PMC9889152; doi:10.1155/2023/1011063)

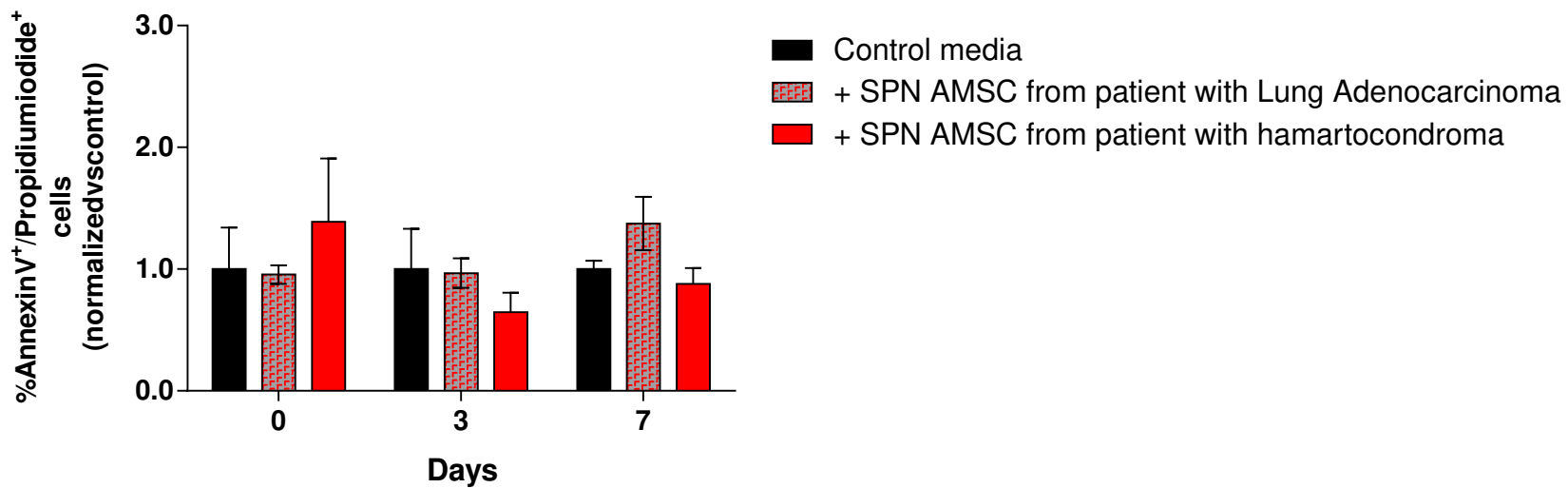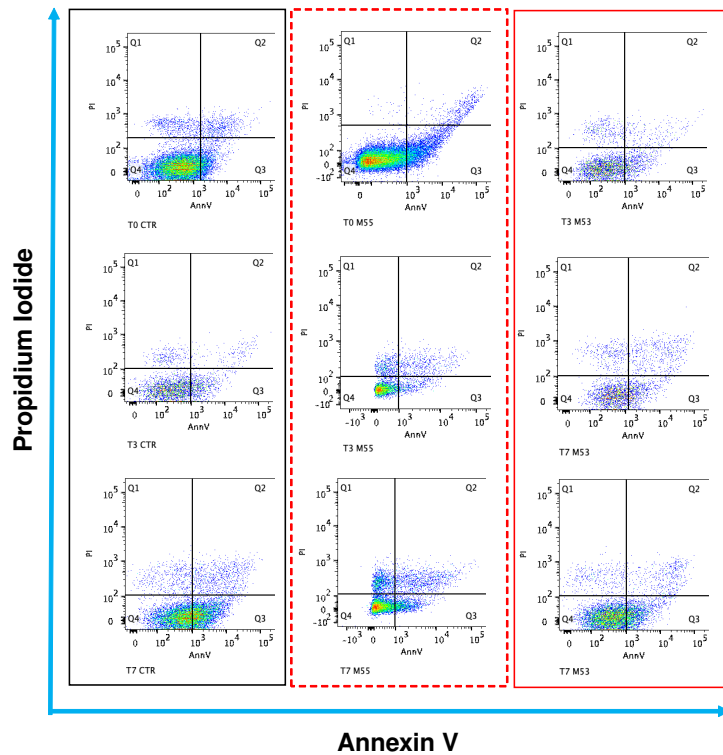

Supplement: Supplementary Materials — Clinical characteristics of adipose tissue-derived MSC samples and the apoptotic rate of the cell cultures treated with MSC-derived supernatants are reported in the supplementary table 1a-b and Figure 1a-b, respectively. [file 1011063.f1.zip › SUpplementary-Figure-1.pdf]
